# CCL2/CCR2 Regulates the Tumor Microenvironment in *HER-2/neu*-Driven Mammary Carcinomas in Mice

**DOI:** 10.1371/journal.pone.0165595

**Published:** 2016-11-07

**Authors:** Xuguang Chen, Yunyue Wang, David Nelson, Sara Tian, Erin Mulvey, Bhumi Patel, Ilaria Conti, Juan Jaen, Barrett J. Rollins

**Affiliations:** 1 Department of Medical Oncology, Dana-Farber Cancer Institute, and Department of Medicine, Brigham & Women’s Hospital, Boston, Massachusetts 02215, United States of America; 2 Harvard Medical School, Boston, Massachusetts 02115, United States of America; 3 ChemoCentryx, Inc., Mountain View, California 94043, United States of America; Universita degli Studi di Milano, ITALY

## Abstract

Chronic inflammation is a hallmark of cancer. Inflammatory chemokines, such as C-C chemokine ligand 2 (CCL2), are often present in tumors but their roles in cancer initiation and maintenance are not clear. Here we report that CCL2 promotes mammary carcinoma development in a clinically relevant murine model of breast cancer. Targeted disruption of *Ccl2* slowed the growth of activated *Her2/neu*-driven mammary tumors and prolonged host survival. Disruption of *Ccl2* was associated with a decrease in the development and mobilization of endothelial precursor cells (EPCs) which can contribute to tumor neovascularization. In contrast, disruption of *Ccr2*, which encodes CCL2’s sole signaling receptor, accelerated tumor development, shortened host survival, and mobilized EPCs. However, pharmacological inhibition of CCR2 phenocopied *Ccl2* disruption rather than *Ccr2* disruption, suggesting that the *Ccr2*^*-/-*^ phenotype is a consequence of unanticipated alterations not linked to intact CCL2/CCR2 signaling. Consistent with this explanation, *Ccr2*^*-/-*^ monocytes are more divergent from wild type monocytes than *Ccl2*^*-/-*^ monocytes in their expression of genes involved in key developmental and functional pathways. Taken together, our data suggest a tumor-promoting role for CCL2 acting through CCR2 on the tumor microenvironment and support the targeting of this chemokine/receptor pair in breast cancer.

## Introduction

Tumor stroma contains a variety of immune cells, endothelial cells, and other mesenchymally derived cell types. Nearly all cancers are infiltrated by inflammatory cells [1–3] which are capable of suppressing or promoting tumor development depending on their phenotypes and abundance. Prominent among these cells are tumor-associated macrophages (TAMs) which can promote tumor growth, angiogenesis, and metastasis in some settings or stimulate anti-tumor immunity or kill tumor cells directly in others [3–6].

Chemokines are mediators of inflammation and immunity which can modulate TAM activity and influence cancer biology [7–9]. CCL2 (or monocyte chemotactic protein-1 (MCP-1)) is a major chemoattractant for monocytes, macrophages, memory T lymphocytes, and endothelial cells [10–12] and directly contributes to the pathogenesis of inflammatory diseases such as atherosclerosis, rheumatoid arthritis and diabetic nephropathy [13, 14]. CCL2 is also associated with the development and progression of several cancer types, including breast, ovarian and prostate [15–19]. Elevated levels of CCL2 in breast cancer biopsies correlate with increased TAM accumulation, more extensive tumor vascularization, and more aggressive clinical behavior [18, 20] and at least a portion of CCL2’s effects may be attributed to its ability to stimulate angiogenesis [21, 22]. Moreover, CCL2 may recruit other effector cells such as Ly-6C^hi^ inflammatory monocytes or mesenchymal stem cells that modulate tumor growth and progression [16, 23]. Recent data suggest that CCL2 may accomplish this through a cascade of chemokine expression involving CCL3 [24]. However, CCL2’s influence on cancer behavior is complex because, in some contexts, it may inhibit tumor growth by attracting tumor-suppressive immune cells [25].

CCL2’s sole signaling receptor is CCR2 [26] and mice carrying targeted disruptions of either *Ccl2* or *Ccr2* have concordant phenotypes in most inflammatory models [13, 27–29]. However, in other settings, the phenotypes of the ligand- and receptor-deleted mice diverge. For example, *Ccl2*^*-/-*^ mice are deficient in T_H_1-biased T cell polarization [27], while *Ccr2*^*-/-*^ mice are T_H_2-deficient [30]. The potential effects of this complex physiology on the behavior of cancers have not been fully explored.

Here, we used a mouse model of breast cancer in which the MMTV LTR drives activated *HER2/neu* (MMTV-*neu*) to investigate how CCL2 and CCR2 affect tumor development. Transgenic MMTV-*neu* mice spontaneously develop aggressive, multifocal mammary carcinomas that mimic many of the characteristics of human breast cancer [31]. We examined the behavior of these mammary carcinomas in mice carrying targeted deletions of *Ccl2* or *Ccr2*, as well as wild type mice treated with a small molecule antagonist of CCR2, and explored the effects of this chemokine ligand/receptor pair on the tumor microenvironment.

## Materials and Methods

### Antibodies

Fluorescent antibodies were purchased from BD Bioscience (Bedford, MA), eBioscience (San Diego, CA), or R&D Systems (Minneapolis, MN) Anti-murine CD31 rat monoclonal (clone Mec13.3) was purchased from Biocare Medical (Concord, CA). The MC-21 antibody against mouse CCR2 was a kind gift from Dr. Detlef Schlöndorff [32].

### Animal models

Wild type Balb/cJ and SCID mice were purchased from Jackson Laboratory (Bar Harbor, ME). MMTV-*neu* mice in an FVB background [31] were backcrossed ten generations into the Balb/cJ background. *Ccl2*^*-/-*^ [29] and *Ccr2*^*-/-*^ [27] (a gift from Israel Charo, University of California at San Francisco) mice in a Balb/cJ background were periodically backcrossed with Balb/cJ mice to reduce genetic drift. In order to place the MMTV-*neu* transgene in the proper background, MMTV-*neu* mice were crossed with *Ccl2*^*-/-*^ or *Ccr2*^*-/-*^ mice. Tumor growth in female *neu*^*+*^ animals was measured weekly using calipers. Protocols for this study were approved by the Institutional Animal Care and Use Committee at Dana-Farber Cancer Institute which is AAALAC accredited, and all animal use was in accordance with the Guide for the Use and Care of Laboratory Animals. Humane endpoints were used in all survival studies. In particular, mice were sacrificed using CO_2_ inhalation followed by cervical dislocation if any of the following endpoints were observed: their tumors reached 2 cm in diameter, their tumors were necrotic, or they were unable to reach food or water. All mice on this study were monitored daily and were administered analgesics or anesthetics if any suffering was observed such as rough hair coat, hunched posture, lethargy, persistent recumbency, labored breathing, or skin breakdown. No unexpected deaths were observed in this study.

### Cell culture

MCF7 and MDA-MB-231 cells were maintained in DMEM (Life Technologies, Grand Island, NY) supplemented with 10% FBS (Life Technologies), and SK-BR-3 cells were maintained in McCoy’s 5A medium (Life Technologies) plus 10% FBS. Human ECFC was purchased from Lonza (Walkersville, MD), and maintained in EBM-2 Basal Medium (Lonza) supplemented with EGM-2 SingleQuot Kit Suppl. & Growth Factors (Lonza). For primary mouse tumor cell isolation, tumors were harvested, cut into small pieces with scissors, and digested with collagenase (Sigma, St. Louis, MO). Cells were filtered through a 40 μm cell strainer, and cultured in DMEM supplemented with 10% FBS.

### CCL2 ELISA

Medium was collected and centrifuged to remove cell debris. Adherent cells were then lysed in 1% NP40 buffer. CCL2 was measured using the OptEIA Human MCP-1 ELISA Set (BD Bioscience). Murine Ccl2 was detected in mouse serum using a preconfigured Bio-Plex mouse cytokine assay (BioRad, Hercules, CA).

*Western blot*: Human mammary carcinoma cells treated with CCL2 neutralizing antibody (R&D Systems) or etoposide (Sigma) were lysed with RIPA buffer (Boston Bioproducts, Ashland, MA), proteins were separated on a 10% Ready Gel precast polyacrylamide gel (Bio-Rad), transferred to a PVDF membrane (Bio-Rad), and probed with a rabbit polyclonal antibody against PARP (Cell Signaling, Danvers, MA). The membrane was then incubated with horseradish peroxidase (HRP)-conjugated secondary antibody and developed using SuperSignal West Pico Chemiluminescent Substrate (Thermo Fisher Scientific, Rockford, IL).

### Cell proliferation assay

Cells were incubated with [^3^H]thymidine at 1 μCi per 500 μL in complete medium containing 10% fetal bovine serum for 24 hrs, and lysed with 0.2 N NaOH for 20 min at room temperature. The lysates were analyzed using a Wallac 1450 MicroBeta TriLux Scintillation Counter (PerkinElmer, Waltham, MA).

### CCX872 treatment

CCX872, a small molecule antagonist of CCR2, was provided by ChemoCentryx (Mountain View, CA). CCX872 is highly specific for CCR2 compared to other chemokine receptors as well as receptors for C5a and fMLP. Its IC_50_ for blocking binding of radiolabeled CCL2 to human CCR2 is 3 nM; its IC_50_ for inhibiting human CCR2-mediated chemotaxis is 32 nM while its IC_50_ for inhibiting murine CCR2-mediated chemotaxis is 69 nM. At a daily dose of 10 mg/kg in mice CCX8782 ameliorated renal dysfunction in diabetic mice to nearly the same extent as 100 mg/kg (Zhenhua Miao, ChemoCentryx, Mountain View, CA, personal communication; see S1 File.). Female MMTV-*neu* littermates were randomly divided into vehicle and treated groups. Mice were given daily subcutaneous injections of 100 μL vehicle (1% HPMC, 0.1% Tween 80) or CCX872 at 2 mg/mL in the same vehicle beginning at 4 weeks of age. Tumors were measured twice weekly using calipers, and volume was averaged.

### Human breast cancer xenograft model

MDA-MB-231 cells were stably transfected with a luciferase reporter gene, and 10^7^ cells in 100 μL PBS containing 5% Matrigel (Life Technologies) were injected into the fourth mammary gland of SCID mice. Injected mice were randomized 1 week after injections, and given subcutaneous injections of isotype antibody or ABN912 antibody against human CCL2 at a dose of 100 or 400 μg [14]. Four more injections were given at an interval of twice weekly. Bioluminescence imaging using a Xenogen instrument (Perkin Elmer) was performed after each injection.

### Tissue microarray (TMA) and immunohistochemistry

Tumors from mice at 60, 80, 100, 120 and 140 days of age were harvested and paraffin-embedded. Five tumor blocks from each genotype at each time point were used to construct a TMA. TMAs were stained for Ki-67, vWF, Mac2, B220, or CD3 with the Vector M.O.M. kit (Vector Laboratories, Burlingame, CA) as directed by the manufacturer. Adjacent sections were stained with isotype controls. TMAs were then imaged, and analyzed quantitatively using Image J software. CD31 staining was performed on slides and quantified by acquiring color images from three fields (0.75 mm x 0.75 mm [2048 pixels x 2048 pixels]) from each slide using a Nikon Eclipse E600 microscope fitted with a SPOT Insight 4.0 camera at 200X. The areas (number of pixels) of vascular structures in each image defined by CD31 staining were quantified and tabulated using ImageJ. Areas of CD31 staining consisting of fewer than 400 pixels were not included in subsequent statistical analysis. Five slides from 5 mice were analyzed for each genotype at each time point (except for *Ccr2*^*-/-*^ at 80 days for which only 4 slides were analyzed). Three images from each slide were analyzed and the average area of CD31 staining per image was determined for each slide. In order to capture small vessel staining, only individual areas of staining with fewer than 10,000 pixels in area were counted.

### Flow cytometry

Peripheral blood was collected from the inferior vena cava of sacrificed mice into EDTA-coated tubes. Bone marrow was flushed from femurs of the same mice in MACS buffer (PBS containing 0.5% BSA and 2 mM EDTA). Spleens were minced, and passed through a 40-micron cell strainer (BD Bioscience) twice. Tumors (usually more than three) from the same mice were minced together with scissors and blades, and were digested in 5 mL RPMI containing 1% collagenase IV (Worthington Biochemical Corporation, Lakewood, NJ) and 1% hyaluronidase (Sigma) at 37°C for 1 hour, and then 10 units/mL DNase I (Worthington) for 15 min. Erythrocytes in blood samples were lysed with RBC Lysis Buffer Solution (eBioscience), and erythrocytes in spleen, bone marrow, and tumor samples were lysed using ACK buffer (Lonza). Erythrocyte-depleted samples were filtered again using a 40-micron cell strainer before staining with fluorescently labeled antibodies in MACS buffer for flow cytometry. When applicable, stained cells were washed with PBS and stained with the viability stain eFluor 506 (eBioscience) in PBS for 30 min before flow analysis. Flow cytometry was performed on an LSR II instrument using proper filters for each fluorophore. Compensation controls were run at the same time using BD CompBeads (BD Bioscience) stained with the same antibodies as those used for cells. Spectral compensations were performed post-acquisition using FlowJo software (Tree Star, Ashland, OR). Gatings were performed based on FMO controls acquired in parallel.

### Monocyte isolation by flow cytometry

Monocytes were defined by the absence of markers for T cells (CD4^–^CD8^–^), B cells (B220^–^), NK cells (NK1.1^–^), dendritic cells (CD11c^–^), neutrophils (Ly-6G^–^) and eosinophils (CCR3^–^) and by the presence of CD11b and Ly-6C. Blood from five wild type, *Ccl2*^*-/-*^, or *Ccr2*^*-/-*^ mice at 14–18 weeks of age was pooled and erythrocytes lysed. Erythrocyte-free blood cells were mixed with the following antibodies at 1:100 dilution (1:10 for CCR3): CD4-PE, CD8-PE, B220-PE, Ly-6G-PE, MHC II-PE (BD Bioscience) and CCR3-PE (R&D Systems), and passed through an LD cell separation column (Miltenyi Biotech, Auburn, CA). Cells in the flowthrough were centrifuged, stained with CD11b-PE antibody at 1:100 dilution, and passed through an MS column (Miltenyi). Positively selected cells were eluted with MACS buffer.

*cDNA Microarray*: Purified monocytes were centrifuged, and total RNA was isolated using RNeasy (QIAGEN, Valencia, CA). RNA quality was assessed using a Nanodrop spectrophotometer and Bioanalyzer. cDNA was synthesized, fluorescently tagged, and hybridized to a Mouse Gene 1.0 ST array (Affymetrix, Santa Clara, CA). Scanned fluorescent images were analyzed using the dChip software. Comparison among samples was conducted using the parameters E/B or B/E >1.2 AND E-B or B-E >100. Gene ontology studies were performed with the GSEA software using the GO Biological Processes of gene ontology (GO) gene sets. Permutation type was set to gene_set and 1000 permutations were conducted. All expression array files are available through GEO. Accession numbers are: GSE88962, GSM2356474, GSM2456475, GSM2456476, GSM2456477, GSM2456478, GSM2456479, GSM2456480, GSM2456481, and GSM2456482.

### Chemotaxis assay

Human ECFC cells were trypsinized and resuspended in 0.1% BSA/RPMI at 10^6^/mL. One hundred μl of cell suspension was added to the upper well of a 24-well Transwell plate with 6.5 mm inserts with pore size of 8.0 μm (Corning Inc, Corning, NY). RPMI (600 μL) containing 0.1% BSA and the indicated concentration of purified CCL2 was added to the lower chamber. Four replicates were included for each treatment, and the assay was terminated at 16 hr. Cells on the bottom of the transwells were fix with methanol at -20°C, and stained with methyl green (0.5% in 0.1M sodium acetate buffer with pH 4.2) at room temperature for 5 min before examination with an inverted microscope. Cells were counted and averaged over 10 randomly chosen fields.

## Results

### Targeted deletions of Ccl2 or Ccr2 have disparate effects on mammary carcinoma behavior in mice

**Fig 1:**
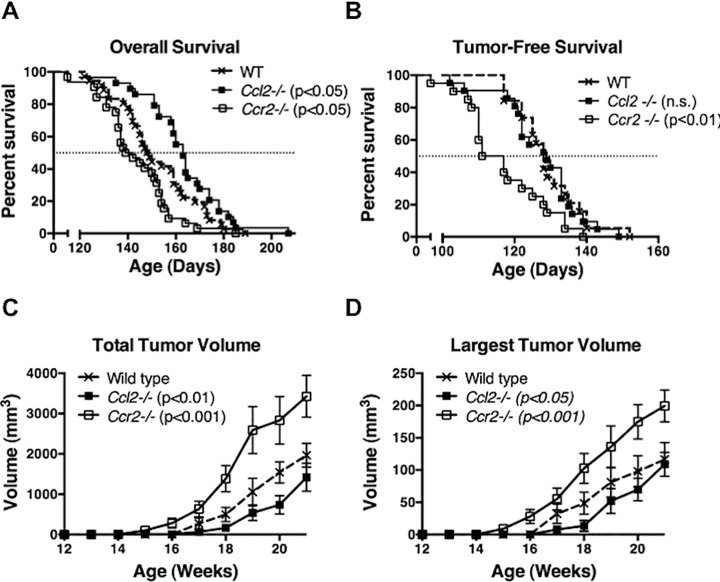
Effect of genetic disruption of *Ccl2* or *Ccr2* on *Her2/neu*-driven mammary carcinoma development. (**A and B**) Overall survival and tumor-free survival of *neu*^*+*^ (n = 36), *neu*^*+*^*Ccl2*^*-/-*^ (n = 29), and *neu*^*+*^*Ccr2*^*-/-*^ (n = 32) mice. Median survivals were compared using Log-rank (Mantel-Cox) test; (**A**) p < 0.05 for both wild type *versus Ccl2*^*-/-*^ and wild type *versus Ccr2*^*-/-*^; (**B**) not significant (n.s.) wild type *versus Ccl2*^*-/-*^, p < 0.01 wild type *versus Ccr2*^*-/-*^. (**C and D**) Growth of *Her2/neu*-driven mammary carcinoma in wild type, *Ccl2*^*-/-*^ and *Ccr2*^*-/-*^ mice measured as total tumor volume in a mouse (**C)** or as the volume of the single largest tumor mass in a mouse **(D)**. Because the stochastic risk of malignant transformation is the same for any mammary epithelial cell in this model, multiple tumor masses were often observed in single mammary glands. Thus the total tumor volume in a mouse (**C**) could exceed the theoretical limit of ten times the single largest tumor mass (**D**) (based on ten mammary glands per mouse). Different strain backgrounds produced no differences in the number of tumors per mouse. Comparisons were made by two-way ANOVA; (**C**) p<0.05 *Ccl2*^*-/-*^ *versus* wild type, p<0.001 *Ccr2*^*-/-*^ *versus* wild type; (**D**) p<0.05 *Ccl2*^*-/-*^ *versus* wild type, p<0.001 *Ccr2*^*-/-*^ *versus* wild type.

To test the effect of CCL2 on the behavior of *HER2/neu*-driven mammary carcinomas, we compared the overall survival of mice carrying the MMTV-*neu* transgene and a targeted deletion of *Ccl2* (*neu*^*+*^*Ccl2*^*-/-*^) to mice carrying the MMTV-*neu* transgene alone (*neu*^*+*^). Fig 1A shows that CCL2-deficient *neu*^*+*^ mice survived significantly longer than *neu*^*+*^ mice. Tumor-free survival i.e., the age at which tumors first appear, was unchanged in *neu*^*+*^*Ccl2*^*-/-*^ mice compared to *neu*^*+*^ mice (Fig 1B) but the rate of tumor growth measured as total tumor burden (Fig 1C) or as the rate of growth of the single largest tumor mass (Fig 1D) was slower in the CCL2-deficient background. Thus, while CCL2 does not affect the age at which tumors first appear, it stimulates their growth once they do appear, hence shortening overall survival. In an attempt to confirm this tumor-promoting effect of CCL2, we developed CCR2-deficient mice carrying the MMTV-*neu* transgene (*neu*^*+*^*Ccr2*^*-/-*^) in the same Balb/c strain background as *neu*^*+*^*Ccl2*^*-/-*^ and *neu*^*+*^ mice. Paradoxically, CCR2 disruption significantly shortened survival (Fig 1A). This correlated with earlier appearance (Fig 1B) and more rapid early growth (Fig 1C and 1D) of tumors in *neu*^*+*^*Ccr2*^*-/-*^ mice.

### Pharmacologic inhibition of CCR2 phenocopies targeted disruption of Ccl2

**Fig 2:**
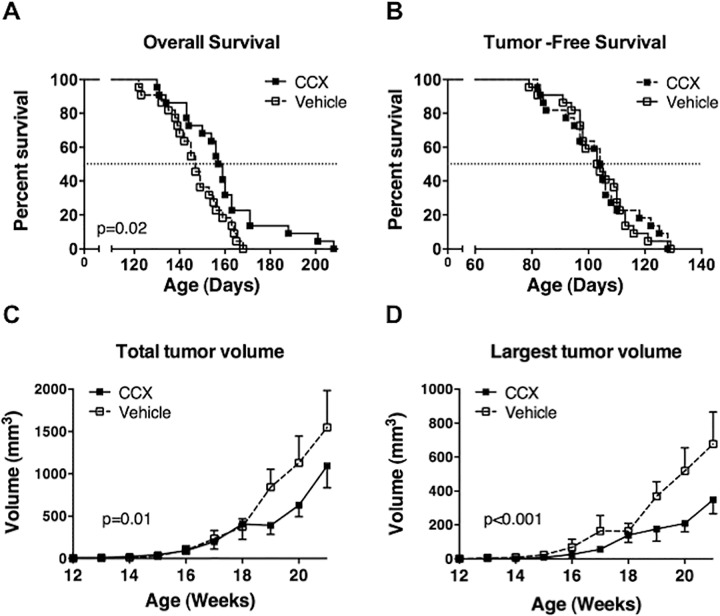
Effect of pharmacologic inhibition of CCR2 on mouse mammary carcinoma development. (**A** and **B**) Overall survival and tumor-free survival of *neu*^*+*^ mice treated with the CCR2 antagonist CCX872 (n = 26) or vehicle (n = 25). Median survivals were compared using Log-rank (Mantel-Cox) test. Mice were sacrificed when the diameter of any single tumor reached 2 cm, if a tumor became necrotic, or if mice were unable to reach food or water (**C** and **D**) Tumor growth in mice treated with CCX872 or vehicle measured as total tumor volume in a mouse **(C)** or as the volume of the single largest tumor mass in a mouse **(D)**. Tumors were measured twice weekly and averaged. Comparisons were made by two-way ANOVA.

The disparate effects of *Ccl2 versus Ccr2* disruption on the behavior of *HER2/neu*-driven mammary carcinomas could be due to the influence of other CCR2 ligands in CCL2-deficient mice or the effects of CCL2 on an as yet unidentified receptor in CCR2-deficient mice. Alternatively, these results could be the consequence of unanticipated alterations that occur in response to genetic disruption of *Ccl2* or *Ccr2*. To test these possibilities, we treated *neu*^*+*^ mice with CCX872, a specific small-molecule antagonist of CCR2. CCX872 prolonged overall survival of *neu*^*+*^ mice without extending their tumor-free survival (Fig 2A and 2B) and suppressed tumor growth (Fig 2C and 2D). Thus the effects of CCX872 are similar to those of *Ccl2* disruption and different from those of *Ccr2* disruption (compare Fig 1). This suggests that the CCL2/CCR2 axis promotes tumor growth in the MMTV-*neu* model and genetic disruption of *Ccr2* promotes tumor growth through other pathways.

### Expression of genes involved in monocyte development and function are significantly altered in Ccr2^-/-^ monocytes

**Fig 3:**
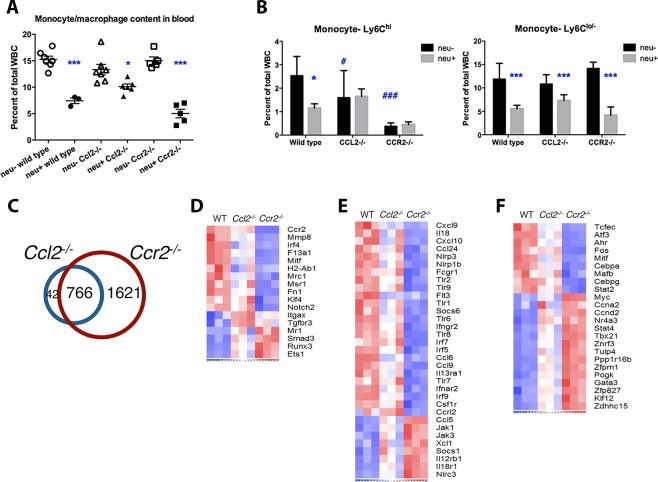
Gene expression profiling of wild type, *Ccl2*^*-/-*^ and *Ccr2*^*-/-*^ monocytes. (**A**) Monocyte proportions in the blood of tumor-free and tumor-bearing wild type, *Ccl2*^*-/-*^ and *Ccr2*^*-/-*^ mice were measured using flow cytometry. (Total leukocyte counts were similar in all three genotypes and unaffected by tumors.) Results were compared using one-way ANOVA and Bonferroni’s multiple comparison test; *, p < 0.05; ***, p < 0.001 comparing *neu*^*-*^ *versus neu*^*+*^ within each genotype. (**B**) Quantitation of Ly-6C^hi^ and Ly-6C^lo/–^monocytes as percentages of total blood leukocytes. Comparisons were made by multiple t-tests. *, p < 0.05; ***, p < 0.001, *neu*^*-*^ *versus neu*^*+*^ within each genotype. #, p < 0.05; ###, p < 0.001, wild type *versus Ccl2*^*-/-*^ or *Ccr2*^*-/-*^ mice. **(C)** Venn diagram of genes differentially expressed between wild type and *Ccl2*^*-/-*^ monocytes, and between wild type and *Ccr2*^*-/-*^ monocytes; 766 genes overlap between the two sets, 42 are unique to the *Ccl2*^*-/-*^ set and 1621 are unique to the *Ccr2*^*-/-*^ set. (**D-F**) Heat maps of genes that characterize Ly-6C^hi^ *versus* Ly-6C^lo/–^monocytes **(B)**, monocyte development and function **(C)**, and transcription factors **(D)**.

Our results raise the possibility that *Ccr2* deletion may produce changes that lead to paradoxical effects on *HER2/neu*-driven tumor growth. To find evidence for such changes, we analyzed gene expression profiles of circulating monocytes isolated from tumor-free wild type, *Ccl2*^*-/-*^, and *Ccr2*^*-/-*^ mice. The proportion of circulating monocytes was the same in all three genotypes (Fig 3A). (Interestingly, the presence of mammary carcinomas reduced the number of circulating monocytes [discussed in more detail, below]). However, monocytes are broadly comprised of two distinct populations: Ly-6C^hi^ inflammatory monocytes are CCR2^+^CX_3_CR1^lo^ while Ly-6C^lo^ monocytes are CCR2^-^CX_3_CR1^hi^ [33, 34], and disruption of *Ccl2* or *Ccr2* specifically suppressed Ly6C^hi^ monocytes (Fig 3B). While *Ccl2* and *Ccr2* disruption affected the abundance of these monocyte subsets similarly, expression array analysis showed a more profound alteration in *Ccr2*^*-/-*^ monocytes than *Ccl2*^*-/-*^ monocytes compared to wild type: *Ccl2*^*-/-*^ monocytes showed significant differences in the expression of 808 genes compared to wild type monocytes (Fig 3C). Although expression of 766 of these genes were also altered in *Ccr2*^*-/-*^ monocytes, these cells showed significant changes in 1621 additional genes suggesting a much more profound departure from the wild type expression profile than *Ccl2*^*-/-*^ monocytes.

**Fig 4:**
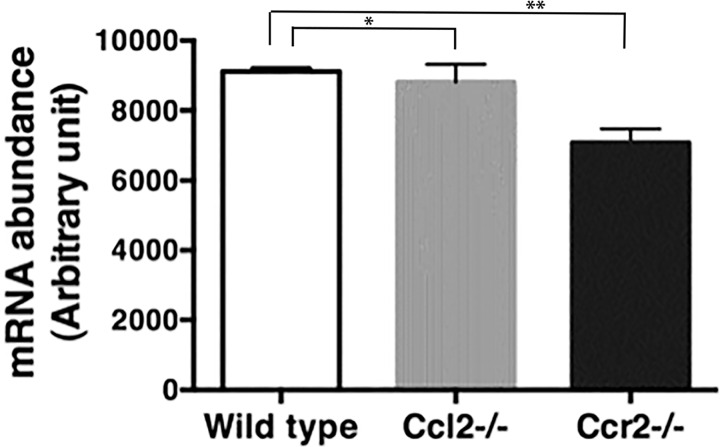
*Csfr1* mRNA is reduced in monocytes from *Ccr2*^*-/-*^ mice. Monocytes were purified from the blood of wild type, *Ccl2*^*-/-*^, and *Ccr2*^*-/-*^ mice (5 mice in each group). mRNA was isolated and mRNA encoding CSF-1R was quantified by PCR. * p > 0.05 by t-test; ** p < 0.05 by t-test.

To explore the implications of these alterations, we examined expression levels of specific genes that are markers for Ly-6C^hi^ and Ly-6C^lo/–^monocytes [34]. Compared to wild type, *Ccl2*^*-/-*^ monocytes showed a bias toward the Ly-6C^lo^ signature, including reduced expression of *Mmp8*, *Irf4*, *Msr1* and *Klf4* and augmented expression of *Tgfbr3*, *Mr1*, *Runx3* and *Ets1*; however, this shift was more pronounced in *Ccr2*^*-/-*^ monocytes (Fig 3D). In addition, *Ccr2*^*-/-*^, but not *Ccl2*^*-/-*^, monocytes expressed lower levels of developmental genes such as *Csf1r* and *Flt3*, as well as genes encoding immune molecules including chemokines, NOD-like receptors, toll-like receptors, interferon regulatory factors, and type I and II interferon receptors (Fig 3D). The reduction in *Csf1r* expression was validated independently by PCR (Fig 4). Finally, wild type and *Ccr2*^*-/-*^ monocytes express different sets of transcription factors that might direct their diverging developmental programs (Fig 3E).

**Fig 5:**
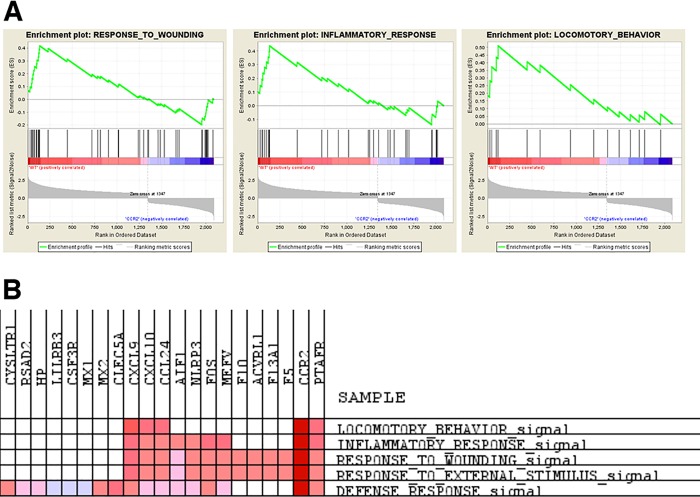
Gene sets enrichment analysis of genes enriched in wild type compared to *Ccr2*^*-/-*^ monocytes. (A) Enrichment plots of wound healing, inflammation and locomotory behavior pathways. (B) Common genes enriched in similar pathways.

Gene sets enrichment analysis (GSEA) indicated that few signaling pathways were altered in *Ccl2*^*-/-*^ monocytes compared to wild type. In contrast, *Ccr2*^*-/-*^ monocytes were markedly deficient in pathways related to host defense: wounding, inflammation, and leukocyte locomotion (Fig 5). Thus *Ccr2*^*-/-*^ monocytes differ from wild type monocytes in important developmental and functional ways. Nonetheless, because of the widespread use of *Ccr2*^*-/-*^ mice we continued to include them in the analyses described below, recognizing that their behavior is dissimilar to postnatal pharmacologic CCR2 inhibition in this setting.

### CCL2 does not directly affect tumor cell proliferation

**Fig 6:**
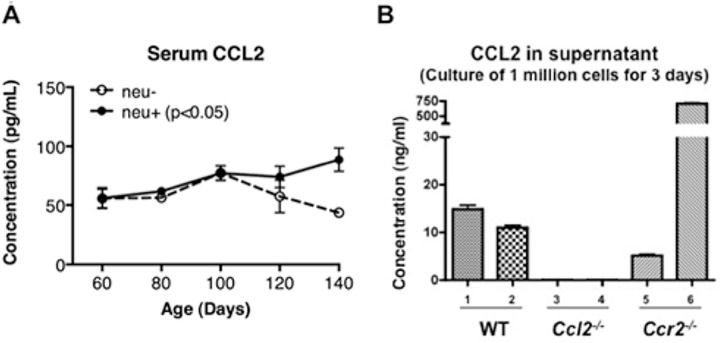
CCL2 production associated with MMTV-neu-driven tumors. (**A**) CCL2 concentrations were measured by ELISA in sera from 4 wild type and 6 *neu*^*+*^ mice at the indicated ages. The difference between wild type and tumor bearing mice was significant by two-way ANOVA (p < 0.05). (**B**) Cell lines were developed from *neu*^*+*^, *neu*^*+*^/*CCL2*^*-/-*^, and *neu*^*+*^/*CCR2*^*-/-*^ mice. CCL2 released into the culture medium by 10^6^ cells over a 72 hr period was measured by ELISA.

**Fig 7:**
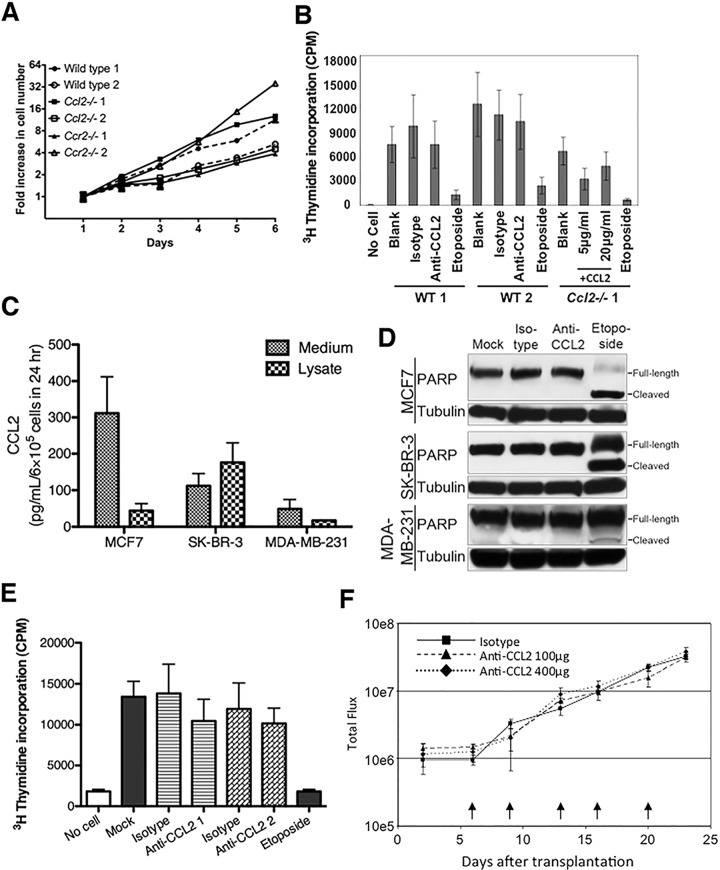
CCL2’s effects on mammary carcinoma cells are non-cell autonomous. (**A**) *In vitro* growth of mammary carcinoma cell lines derived from MMTV-*neu*-induced tumors in wild type, *Ccl2*^*-/-*^, and *Ccr2*^*-/-*^ mice. Two representative cell lines were tested from each genotype. (**B**) Neutralization of CCL2 in cultures of wild type murine tumor cells or addition of exogenous CCL2 to *Ccl2*^*-/-*^ tumor cells did not affect cell proliferation *in vitro* as measured by ^3^H-thymidine incorporation. Etoposide was used as positive control for growth inhibition. (**C**) Concentration of CCL2 protein in medium or cell lysates of cultured MCF7, SK-BR-3 or MDA-MB-231 human breast cancer cell lines determined by ELISA. (**D**) Immunoblot detection of full-length and cleaved poly(ADP-ribose) polymerase (PARP) in lysates from MCF7, SK-BR-3 or MDA-MB-231 cells treated with CCL2-neutralizing antibody. Etoposide was used as a positive control for PARP cleavage. Blots were stripped and reblotted for tubulin as a loading control. (**E**) MDA-MB-231 cells were treated with two different CCL2-neutralizing antibodies, and cell proliferation was measured by ^3^H-thymidine incorporation. (**F**) Luciferase-expressing MDA-MB-231 cells were injected into the mammary fat pads of SCID mice. Mice were treated with ABN912, a neutralizing anti-human CCL2 antibody, or isotype control. Arrows indicate time of antibody treatment. Tumor growth was followed by bioluminescence imaging.

Both tumor-free and tumor-bearing mice have detectable amounts of CCL2 in serum although tumor-bearing mice have higher levels as their tumors progress after 120 days of age (Fig 6A). Cell lines derived from *MMTV-neu* carcinomas in wild type and *Ccr2*^*-/-*^ backgrounds secrete CCL2 in culture (Fig 6B). These observations suggest the possibility that CCL2 might promote tumor growth through a direct effect on tumor cells. However, tumor cells from *neu*^*+*^, *neu*^*+*^*Ccl2*^*-/-*^, and *neu*^*+*^*Ccr2*^*-/-*^ mice grow at similar rates in culture (Fig 7A), and adding neutralizing CCL2 antibodies to wild type tumor cells or exogenous CCL2 to *Ccl2*^*-/-*^ tumor cells did not affect their proliferation (Fig 7B). These observations extended to human breast cancer cell lines: neutralization of CCL2 did not induce apoptosis or inhibit proliferation (Fig 7C–7E). Similarly, antibody blockade of human CCL2 in a mouse xenograft model did not alter the growth of transplanted mammary tumors (Fig 7F), suggesting that autocrine CCL2 does not contribute to tumor growth in this model. (However, since CCL2 may be produced by non-malignant stromal cells in mammary cancers *in situ*, this experiment does not rule out a direct effect of paracrine murine CCL2.)

### Effects of CCL2 and CCR2 deletion on TAM accumulation

**Fig 8:**
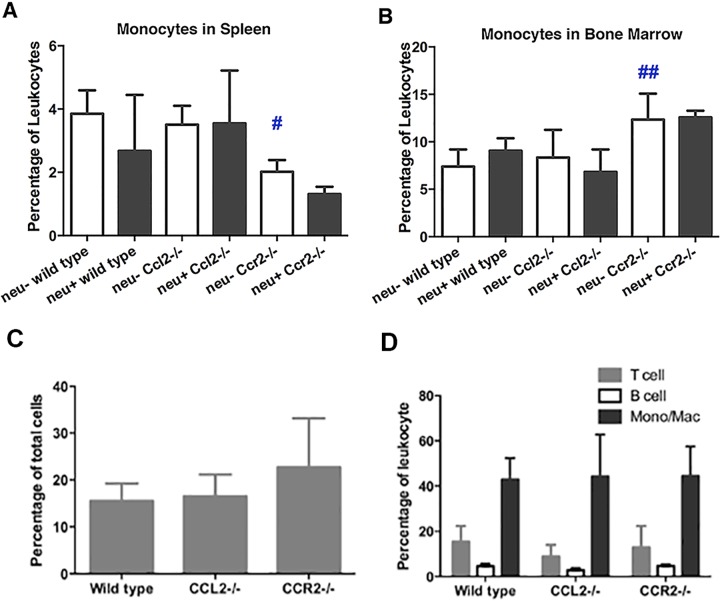
Effect of mammary tumors, *Ccl2* disruption, and *Ccr2* disruption on monocyte accumulation in spleen, bone marrow, and tumors. (**A** and **B**) Proportions of monocytes in the spleen (**A**) and bone marrow (**B**) of tumor-free and tumor-bearing wild type, *Ccl2*^*-/-*^ and *Ccr2*^*-/-*^ mice were measured by flow cytometry. Comparisons were made by one-way ANOVA and Bonferroni’s multiple comparison test. #, p < 0.05; ##, p < 0.01 wild type *versus Ccl2*^*-/-*^ or *Ccr2*^*-/-*^ mice. (**C**) Total leukocyte content of tumors was determined as the percentage of CD45^+^ cells. (**D**) Proportions of tumor-infiltrating T cells, B cells, and monocytes/macrophages were determined by flow cytometry using antibodies against CD90^+^, B220^+^, and CD11b^+^, respectively.

As noted above (Fig 3A), tumor-bearing animals had lower numbers of circulating monocytes than tumor-free animals. To better understand the basis for the reduced numbers of blood monocytes in tumor-bearing mice, we examined monocyte reservoirs. The presence of tumors did not influence the number of monocytes in the spleen or bone marrow (Fig 8A and 8B) suggesting that mammary carcinomas do not cause splenic pooling or suppression of monocyte production. This inference is supported by unchanged levels of M-CSF in tumor-bearing animals (not shown). CCR2 deficiency reduced the number of monocytes in the spleen while increasing their number in bone marrow consistent with the marrow emigration defect previously reported in *Ccr2*^*-/-*^ mice [35]. However, neither CCL2 nor CCR2 deficiency had any effect on the accumulation of total leukocytes in tumors (Fig 8C) or TAMs (Fig 8D). Further experimentation will be required in order to determine if CCL2 or CCR2 deficiency influences the phenotype of these TAMs. Thus the most likely explanation for the tumor-driven decrease in circulating monocytes is that circulating monocytes are being destroyed in the presence of tumors through as yet unknown mechanisms.

### Differential effects of CCL2 and CCR2 deletion on endothelial progenitor cells

**Fig 9:**
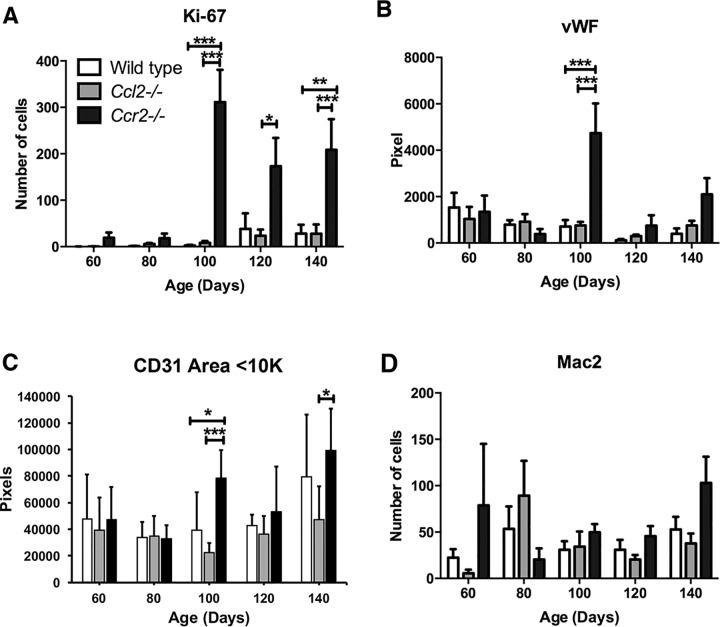
Measurement of tumor-associated leukocytes and endothelial cells. Tissue microarrays were prepared from MMTV-*neu*-driven tumors in wild type, *Ccl2*^*-/-*^, and *Ccr2*^*-/-*^ mice at 60, 80, 100, 120 and 140 days of age. Four or five mice were included per data point. Tissue microarray slides treated with fluorescent antibodies against (**A**) Ki-67, (**B**) vWF, (**C**) CD31, and (**D**) Mac2 and imaged by fluorescence or immunohistochemistry. Quantified stains were compared using one-way ANOVA. *, p < 0.05; **, p < 0.01; ***, p < 0.001.

Because tumor growth can be affected by blood supply, we tested whether angiogenesis was altered in the various genetic backgrounds by examining tissue microarrays and slides using tumors isolated from *neu*^*+*^, *neu*^*+*^*Ccl2*^*-/-*^, and *neu*^*+*^*Ccr2*^*-/-*^ mice at different ages. Consistent with host survival and tumor growth in vivo (Fig 1), tumors from wild type and *Ccl2*^*-/-*^ mice showed similar patterns of gradually increasing Ki-67 staining of tumor cells (identified morphologically) while *neu*^*+*^*Ccr2*^*-/-*^ tumors showed much higher levels of Ki-67 staining starting abruptly at 100 days (Fig 9A). This was also the age at which increases in von Willebrands factor (vWF) and CD31 appeared in *Ccr2*^*-/-*^ tumors (Fig 9B and 9C) suggesting that these tumors underwent an angiogenic switch at that time. Notably, CD31 staining density continued to increase in *Ccr2*^*-/-*^ mice through 140 days of age. As seen in the cells eluted from tumors (Fig 8D), there were no significant differences among the various genotypes in the number of tumor-associated macrophages at most time points (Fig 9D).

**Fig 10:**
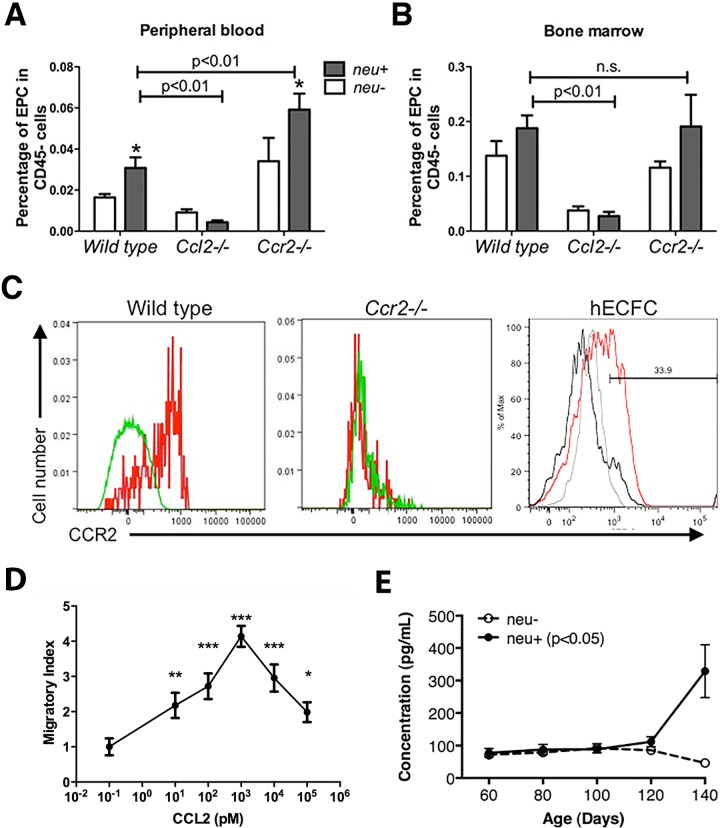
Effects of mammary tumors, *Ccl2* disruption, and *Ccr2* disruption on endothelial progenitor cell (EPC) development and mobilization. (**A** and **B**) EPCs in peripheral blood (**A**) and bone marrow (**B**) of tumor-free and tumor-bearing wild type, *Ccl2*^*-/-*^, and *Ccr2*^*-/-*^ mice were measured as the proportion of circulating CD45^-^ cells. Total CD45^-^ cell numbers were similar in all three genotypes with and without tumors. Comparisons were made by multiple t-tests. *, p < 0.05, *neu*^*-*^ vs. *neu*^*+*^. (**C**) Expression of CCR2 by mouse EPC and human endothelial colony-forming cells (hECFC). Left panel: red histogram, anti-CCR2; green histogram, isotype control. Middle panel: red histogram, anti-CCR2; green histogram, isotype control. Right panel: red histogram, anti-CCR2, grey histogram, isotype control; black histogram, unstained cells. (**D**) Chemotaxis of human endothelial colony-forming cells (hECFC) in response to purified human CCL2. Cultured ECFCs were plated in the upper well of a Boyden chamber, and increasing concentrations of CCL2 were added to the lower chamber. Migrated cells were stained with methyl green, and ten randomly chosen fields were counted. Migratory index was normalized to the number of migrated cells in medium only (artificially set to 10^−1^ pM on the plot). Comparisons were made by t-tests. *: p<0.05; **: p<0.01; ***: p<0.001. (**E**) Serum VEGF levels are higher in tumor-bearing animals. VEGF concentrations were measured by ELISA in sera from 4 wild type and 6 *neu*^*+*^ mice at the indicated ages. The difference between wild type and tumor bearing mice was significant by two-way ANOVA (p < 0.05).

Tumor angiogenesis can be accomplished, in part, by recruitment of endothelial progenitor cells (EPCs) [36]. Therefore, we examined whether the presence of mammary carcinomas affects the development and deployment of CD45^–^CD117/c-Kit^+^Flk1/Vegfr2^+^ EPCs and whether CCL2 and CCR2 play a role. The presence of tumors in wild type mice doubled the proportion of EPCs in peripheral blood (Fig 10A). However, EPCs in bone marrow were not significantly increased (Fig 10B) suggesting that the primary effect of mammary cancers is to increase EPC mobilization without enhancing their production. Deletion of *Ccr2* increased the proportion of circulating EPCs in wild type mice and the presence of mammary tumors nearly doubled this proportion (Fig 10A). Both of these effects, increased basal EPC numbers and tumor-induced increases in EPCs, appeared to be the result of mobilization since *Ccr2* deletion did not alter bone marrow EPCs (Fig 10B). Strikingly, deletion of *Ccl2* reduced circulating EPCs by 50% and bone marrow EPCs by 80%, and neither proportion increased in the presence of tumors (Fig 10A and 10B). This suggests that CCL2 is required both for the development of EPCs and for their mobilization by mammary cancers. Murine EPCs express CCR2 [37] as do their human counterparts, human endothelial colony forming cells (hECFCs), (Fig 10C) and hECFCs respond chemotactically to CCL2 in vitro (Fig 10D). The mechanism of EPC recruitment into tumors may also depend on VEGF since circulating VEGF levels rise in tumor bearing mice after 100 days of age (Fig 10E).

## Discussion

We have demonstrated that targeted disruption of *Ccl2* and pharmacologic inhibition of CCR2 both delay the growth of mammary tumors and prolong survival of mice carrying the MMTV-*neu* transgene. These results suggest that CCL2-dependent CCR2 signaling promotes the development and growth of endogenous murine mammary carcinomas induced by *HER2/neu*, a driver oncogene in 20% of human breast cancers [38]. Rather than exerting its effects directly on mammary carcinoma cells by enhancing proliferation or survival as described in other models [19, 39], CCL2 molds the tumor microenvironment to promote tumor progression. Specifically, although tumors in CCL2-deficient mice have the same total number of TAMs as tumors in wild type mice, *Ccl2* disruption is associated with a profound reduction in the numbers of endothelial precursor cells (EPCs) in the bone marrow and circulation which may suppress tumor angiogenesis.

These murine modeling results are consistent with clinical studies. For example, CCL2 concentrations in human breast cancers are higher than in normal breast tissue [20] and serum CCL2 is higher in breast cancer patients than in healthy controls [18]. Elevated serum CCL2 is associated with increased tumor macrophage infiltration, angiogenesis, and shortened survival [18].

Our results are also consistent with other mouse models in which CCL2 attracts tumor-promoting macrophages to metastatic sites of mammary carcinomas driven by polyoma middle T antigen [16, 24]. However, tumor-promoting macrophages are not present in primary cancer sites in that model. This contrasts with the MMTV-*neu* primary tumors reported here, in which TAMs are present in the primary tumor, and this discrepancy is likely due to differences between the transgenes used in the models; tumor-associated inflammation is more pronounced in polyoma middle T-driven tumors than in those driven by *HER2/neu* [40]. However, similar to the MMTV-*neu* model, anti-CCL2 treatment decreases the growth rate and microvessel density of primary polyoma middle T-driven tumors [20]. Another difference between these models is that the polyoma middle T tumors produce frequent pulmonary metastases while this is an extremely rare occurrence in MMTV-*neu* mice, highlighting the fact that our observations are restricted to the effects of CCL2 and CCR2 on primary tumors [41]. Although we did not document M2 polarization of TAMs in our model, our results could be consistent with models in which CCL2 expression drives M2 polarization of TAMs in melanoma xenografts [42] and in human peripheral blood mononuclear cells [43]. However, the most profound effect of CCL2/CCR2 appears to be on endothelial precursor cells (see below).

Paradoxically, we observed that targeted disruption of *Ccr2*, which encodes the only high affinity signaling receptor for CCL2, promotes *HER2/neu*-driven tumor growth and shortens overall survival. Although the phenotypes of CCL2- and CCR2-deficient mice are concordant in models of inflammatory disease such as atherosclerosis or experimental allergic encephalitis [13, 28, 44–46], they differ in other settings. For example, *Ccl2*^-/-^ mice are deficient in T_H_2 T lymphocyte function [30] while *Ccr2*^*-/-*^ mice are T_H_1-deficient [27]. Speculative explanations for these differences have included the possibility that the other high affinity ligands of CCR2 such as CCL7, CCL8, CCL12, and CCL13 may signal through CCR2 in *Ccl2*^*-/-*^ mice while all such signaling would be abrogated in *Ccr2*^*-/-*^ mice. However, the levels of these chemokines have generally been reported to be unchanged in *Ccl2*^*-/-*^ mice [29, 44], although there is one report of decreased CCl7 expression [47]. Alternatively, CCL2 might signal through an as yet unidentified receptor in *Ccr2*^*-/-*^ mice.

Here, however, we considered the additional possibility that genetic disruption of *Ccl2* or *Ccr2* might lead to asymmetric secondary changes in gene expression which could produce divergent phenotypes. In fact, even though *Ccl2*^*-/-*^ monocytes showed differences in the expression of several genes compared to wild type monocytes, *Ccr2*^*-/-*^ monocytes differed in the expression of three times as many. Furthermore, GSEA showed that CCR2-deficient monocytes diverged more profoundly from wild type monocytes in functional and developmental pathways than did CCL2-deficient monocytes. These results suggest that the degree of phenotypic divergence from wild type is far greater in *Ccr2*^*-/-*^ mice than *Ccl2*^*-/-*^ mice. The potential for this difference to produce artifacts is highlighted by the fact that highly specific pharmacologic inhibition of CCR2 phenocopies *Ccl2* disruption rather than *Ccr2* disruption. The MMTV-neu model may specifically highlight this discrepancy since the phenotypes of *Ccl2*^*-/-*^ and *Ccr2*^*-/-*^ mice are similar in polyoma middle T antigen-driven mammary carcinoma [48].

Our results also reveal the effects that actively growing carcinomas have on monocyte development, trafficking, and physiology. Wild type mice with *HER2/neu*-driven tumors have many fewer circulating monocytes than tumor-free animals. Because these tumors have no effect on the number of monocytes in bone marrow, spleen, or tumors themselves, we infer that they influence monocyte survival in the periphery. Such a process might be expected to be influenced by the CCL2/CCR2 axis and, in fact, *Ccl2* disruption appears to reverse some of the decrease in monocyte numbers induced by the presence of tumors (see Fig 3A) although we have not identified the mechanism. We also found that CCR2 deficiency leads to accumulation of monocytes in the bone marrow, an effect first described by Serbina and Pamer in a Listeria model [35]. However, this may not be a direct result of CCL2 signaling through CCR2 since monocytes do not accumulate in the bone marrow in *Ccl2*^*-/-*^ mice. This is consistent with the much more profound effect of *Ccr2* disruption than *Ccl2* disruption on retention of bone marrow monocytes in the Listeria model [49]. This may be another example of the developmental influence of CCR2 loss leading to a discrepancy between the phenotypes of *Ccl2*^*-/-*^ and *Ccr2*^*-/-*^ mice.

Although the highly aggressive behavior of tumors in *Ccr2*^*-/-*^ mice appears to be a consequence of more than just loss of CCR2 signaling, the tumors in these mice provided a clue about a mechanism by which intact CCL2/CCR2 signaling influences tumor progression. The sudden increase in neovascularization in the tumors of CCR2-deficient mice suggests that they underwent an angiogenic switch around 100 days of age. This led us to examine endothelial progenitor cells (EPCs), a minor population of bone marrow-derived circulating cells that have endothelial markers and are recruited to both primary tumor and pre-metastatic niches to initiate tumor neovascularization [36, 37]. We found that the number of circulating EPCs increased in tumor-bearing mice compared to tumor-free mice and that disruption of *Ccl2* and *Ccr2* had profound effects on circulating EPC numbers: EPCs were greatly reduced in *Ccl2*^*-/-*^ mice and increased in *Ccr2*^*-/-*^ mice. In contrast to CCL2’s effects on monocytes, which seems restricted to mobilization or trafficking, CCL2 deficiency greatly reduced the number of EPCs in the bone marrow. Furthermore, the stimulation of EPC generation and mobilization by tumors was abrogated in the absence of CCL2. These observations suggest that CCL2 directly influences both the development and the mobilization of EPCs [37]. We propose a model in which (1) CCL2 stimulates EPC development in the marrow, (2) mammary carcinomas mobilize EPCs in a CCL2-dependent manner, and (3) EPC migration into tumors is mediated by other factors such as VEGF which rises in the serum of tumor-bearing mice.

A potential limitation of our model is that it is driven by an activating mutation of rat *HER2/neu* which leads to much more aggressive tumor behavior than the amplification seen most commonly in human breast cancer [38]. On one hand, this might lead to differences in the way CCL2/CCR2 affects murine tumors compared to human tumors; on the other hand, this aggressive model may mask to some extent the effects of CCL2/CCR2. The impact of this chemokine ligand/receptor pair might actually be more profound in slower growing, non-mutated *HER2/neu*-amplified tumors. Still, recent reports of activating mutations of *HER2/neu* in breast cancer support the relevance of the murine model to human disease [50]. Taken together, our data support the validity of CCR2 as a therapeutic target in breast cancer.

## Supporting Information

S1 File Dosing and efficacy of CCX872 in mice. An abstract from ChemoCentryx presented at the 2012 meeting of the American Diabetes Association. It describes the dose of CCX872 which was effective in ameliorating renal failure in diabetic mice. It also provides evidence for the specificity of CCX872 for the receptor CCR2.
(PDF)
