# Supplementary material for: CCL2/CCR2 Regulates the Tumor Microenvironment in HER-2/neu-Driven Mammary Carcinomas in Mice
Source: PLoS One. 2016 Nov 7;11(11):e0165595. doi: 10.1371/journal.pone.0165595 (PMC5098736; doi:10.1371/journal.pone.0165595)
Supplement: S1 File — An abstract from ChemoCentryx presented at the 2012 meeting of the American Diabetes Association. It describes the dose of CCX872 which was effective in ameliorating renal failure in diabetic mice. It also provides evidence for the specificity of CCX872 for the receptor CCR2. (PDF) [file pone.0165595.s001.pdf]

Poster #517-P

CCR2 Inhibition Improves Renal Function in Diabetic BKS db/db Mice

Timothy J. Sullivan, Zhenhua Miao, Bin N. Zhao, Robert D. Berahovich, Jay P. Powers, Linda Ertl, Juan C. Jaen, and Thomas J. Schall; ChemoCentryx, Inc., Mountain View, CA, United States

Introduction

Background

- The chemokine receptor CCR2 has been implicated in the recruitment of blood monocytes into kidney in response to hyperglycemia and hypertension. This infiltration is mediated by upregulated production of MCP-1 (CCL2) by glomerular and tubular cells
- Glomerular cells are thought to also upregulate CCR2 in response to the same diabetic stimuli.
- CCX140-B is an orally-administered specific CCR2 antagonist currently being tested in two Phase 2 clinical trials in patients with diabetic nephropathy (DN). The larger of the ongoing Phase 2 clinical trials with CCX140 targets at least 135 DN subjects with moderate to severe proteinuria, reduction of which will be a key study outcome. Placebo or CCX140-B (5 or 10 mg/day) will be administered for at least 12 weeks.
- Given the high specificity of CCX140-B for human versus mouse CCR2, preclinical data in a mouse model of DN was generated using CCX872, a new CCR2 antagonist with increased potency on mouse CCR2.

Materials and Methods

- Male BKS db/db mice (Jackson Labs C57BLKS/J-leprdb/leprdb), 28 weeks of age, were assigned to receive either vehicle control or various doses of CCX872. At study initiation, mice were extremely diabetic (fasting plasma glucose 500-600 mg/dL) and proteinuric (UAER 700-800 µg/day).
- CCX872 is orally bioavailable but, unlike larger animals, its half-life in mice is short. In order to maintain constant plasma levels of compound, it was dosed subcutaneously in aqueous 1% HPMC/0.1% tween-80. Doses of either 10 or 100 mg/kg were administered daily for 13 weeks.
- Glomerular Filtration Rate was determined during week 13 of the study by measuring inulin-FITC clearance after iv administration.
- At sacrifice, one kidney from each animal was excised and fixed in 10% formalin for 48 hours. After embedding in paraffin, 3 µm-thick sections were stained with the appropriate antibody (WT-1; F4/80, 7/4; anti-CD3) for immunohistochemistry.

CCX872 Is Highly Selective for CCR2 Relative to All Other Chemokine and Chemotactic Receptors Tested

Receptor	Assay	CCX872 Potency
CCR1 (Monocytes, CKβ-8)	Calcium Flux	No effect @ 10 µM
CCR2 (Monocytes, MCP1)	Serum Migration	A_2 ~ 10 nM
CCR3 (293CCR3, Eotaxin)	Calcium Flux	No effect @ 10 µM
CCR4 (Lymphocytes, MDC)	Calcium Flux	No effect @ 10 µM
CCR5 (L1.2-CCR5, MIP1β)	Serum Migration	A_2>10 µM
CCR6 (Lymphocytes, MIP3α)	Calcium Flux	No effect @ 10 µM
CCR7 (Lymphocytes, MIP3β)	Calcium Flux	No effect @ 10 µM
CCR8 (293CCR8, I309)	Calcium Flux	No effect @ 10 µM
CCR9(MOLT-4, H^3CCX8477)	Radioligand Binding	IC_{50}>20 µM
CCR10 (293-CCR10, CCL28)	Calcium Flux	No effect @ 10 µM
CCR12 ((Neutrophils, SHAAGtide)	Calcium Flux	No effect @ 10 µM
CXCR1 (Neutrophils, IL8)	Calcium Flux	No effect @ 10 µM
CXCR2 (Neutrophils, IL8)	Calcium Flux	No effect @ 10 µM
CXCR3 (Lymphocytes, ITAC)	Calcium Flux	No effect @ 10 µM
CXCR4 (Lymphocytes, SDF1α)	Calcium Flux	No effect @ 10 µM
CXCR5 (BaFCXCR5, BCA)	Serum Migration	A_2>10 µM
CXCR6 (IL2-Lym., MIP3α)	Calcium Flux	No effect @ 10 µM
CXCR7 (435CXCR7, I^{125}SDF1α)	Radioligand Binding	IC_{50}>10 µM
C3aR (Neutrophils, C3α)	Calcium Flux	No effect @ 10 µM
C5aR (Neutrophils, C5α)	Calcium Flux	No effect @ 10 µM
FPR1 (Neutrophils, fMLP)	Calcium Flux	No effect @ 10 µM
Duffy (Whole Blood, I^{125}MCP1)	Radioligand Binding	IC_{50}>20 µM

CCX872 Is a Potent and Selective Antagonist of Human and Mouse CCR2

Assay	Cell Type	IC_{50}
Radioligand Binding (buffer, ^{3}H-7942, IC_{50}, nM)	293-hCCR2	3 nM
Chemotaxis (100% serum, MCP-1, A_2, nM, pH=7.2)	L1.2-hCCR2	32 nM
	Human Monocytes	15 nM
Mouse CCR2 (WEHI cells; Chemotaxis; A_2, nM)	buffer	69 nM
	plasma	1200 nM
CYP inhibition (IC_{50})	1A2	>30 mM
	2C9	>10 mM
	2D6	>30 mM
	2C19	>30 mM
	3A4	>10 mM
Time-Dependent CYP Inhibition (10 µM)	3A4	Clean
	2C9	
CYP Induction (10 µM)	1A2	Clean
	3A4	

Calculated Receptor Coverage of CCX872 in BKS *db/db* Mice

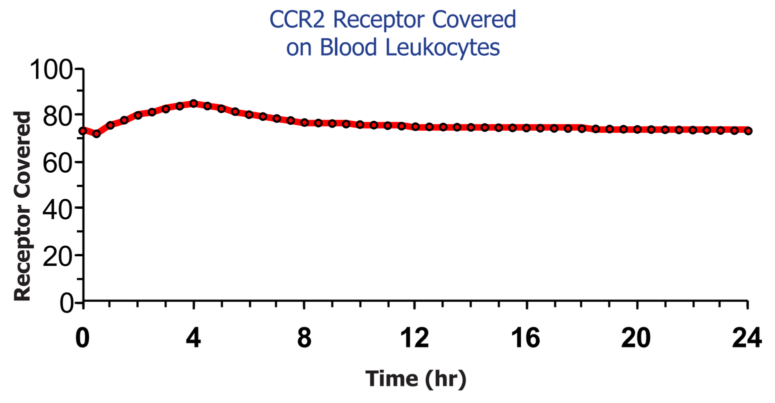

- Based on the PK profile of CCX872 in mice and its potency for mouse CCR2, the high dose of CCX872 provides an extent of receptor coverage in mice which is somewhat lower than that achieved with the clinical compound CCX140-B in humans.

CCX872 Significantly Reduces Daily Urinary Albumin Excretion in Old BKS *db/db* Mice

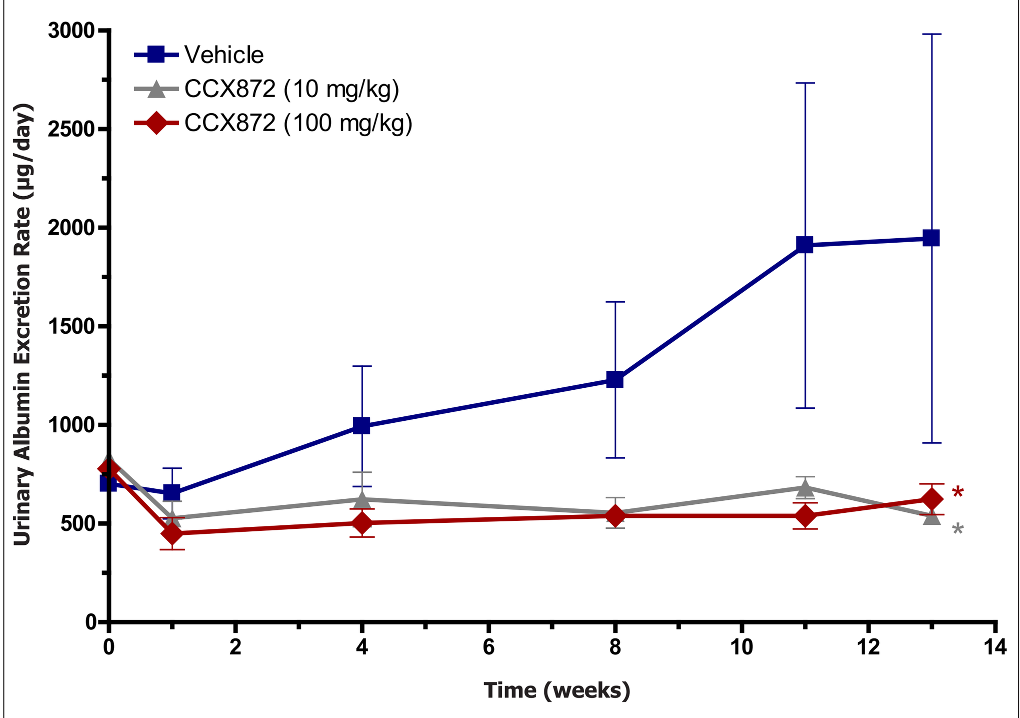

*p<0.05 vs. vehicle

CCX872 Significantly Reduces Daily Urinary Albumin Excretion in BKS *db/db* Mice (Change from Baseline)

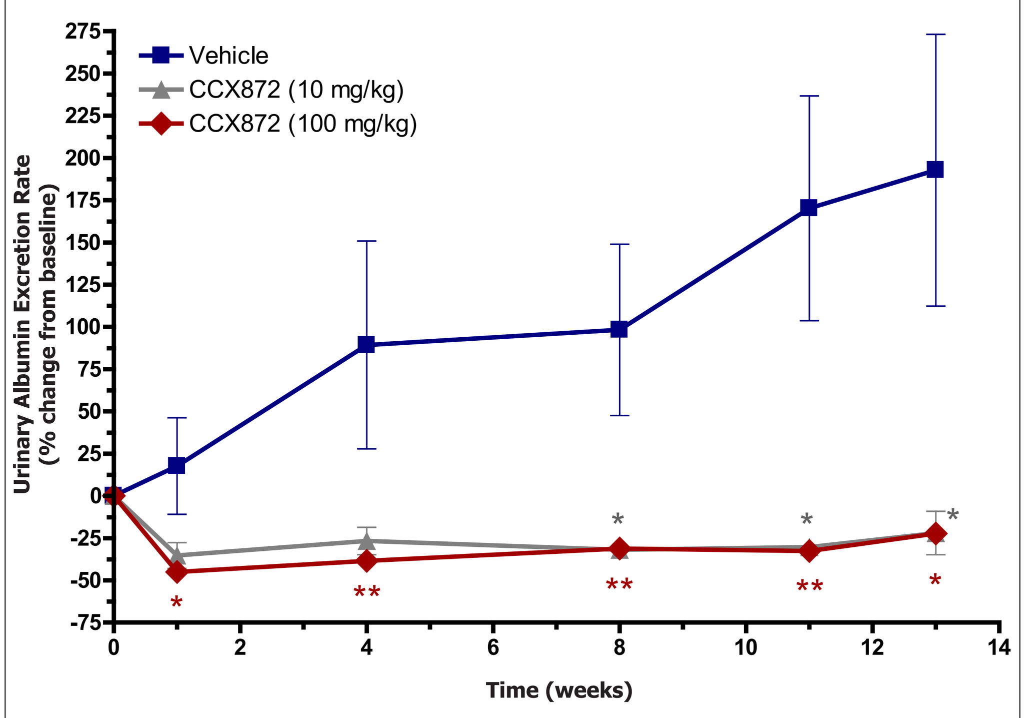

*p<0.05 vs. vehicle; **p<0.005 vs. vehicle

CCX872 Significantly Reduces Urinary Albumin:Creatinine Ratio (ACR) in Old BKS *db/db* Mice

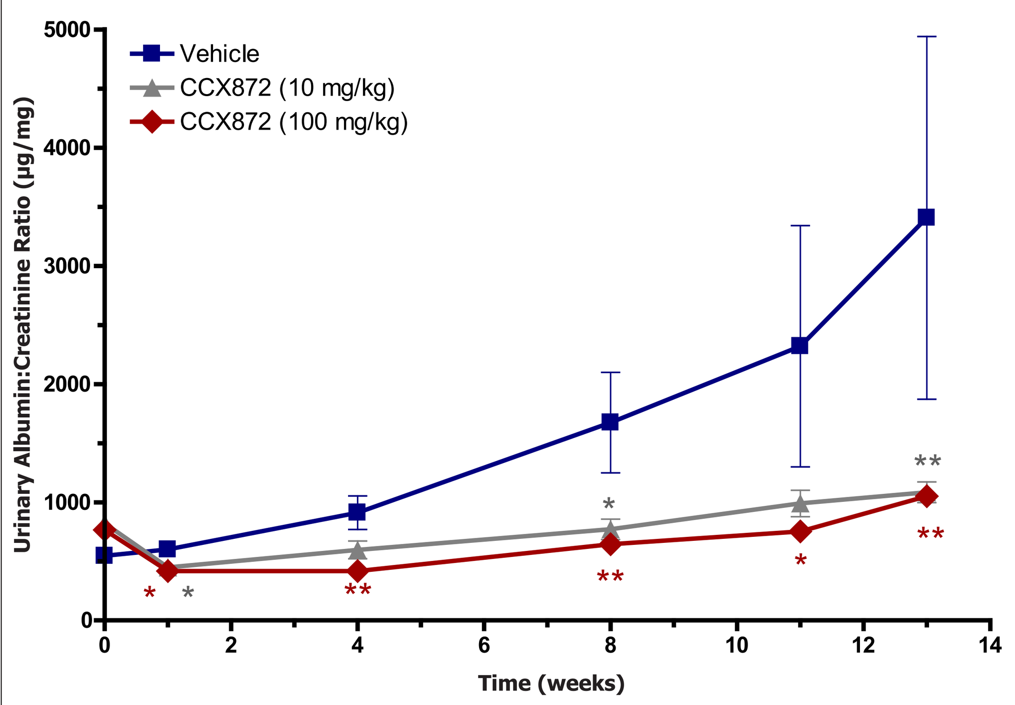

*p<0.05 vs. vehicle; **p<0.005 vs. vehicle

CCX872 Significantly Reduces Urinary ACR in Old BKS *db/db* Mice (Change from Baseline)

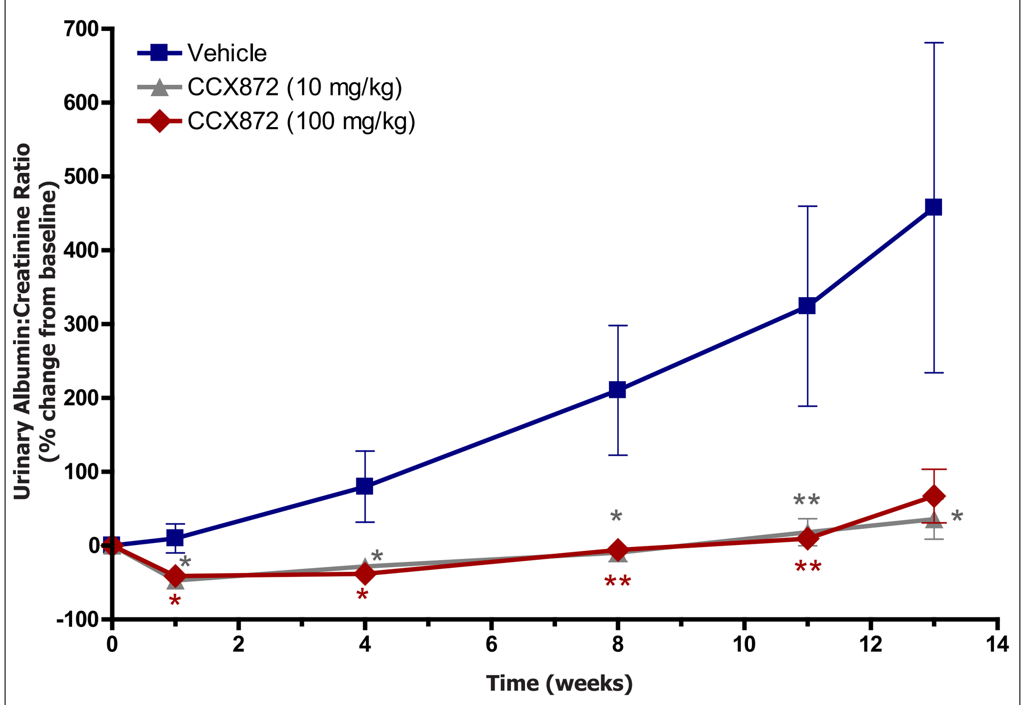

*p<0.05 vs. vehicle; **p<0.005 vs. vehicle

Effects of CCX872 on Glomerular Hyperfiltration, Glomerular Volume, and Glomerular Podocyte Density

CCX872 Reduces Glomerular Hyperfiltration in Old BKS *db/db* Mice

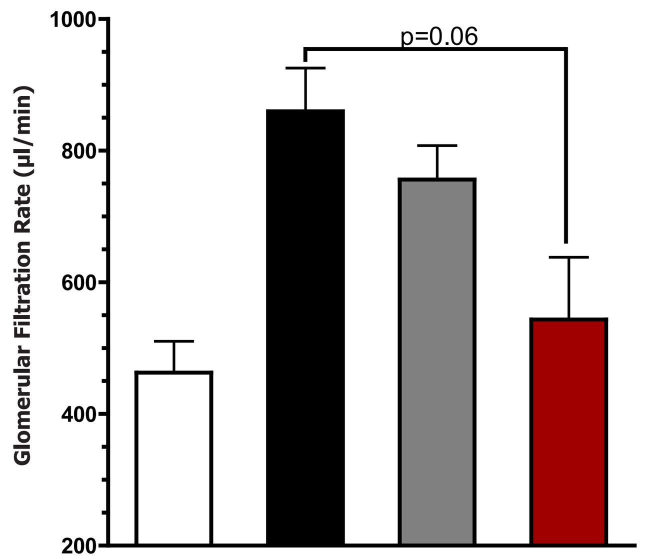

- Glomerular Filtration Rate (GFR) determined using inulin-FITC plasma clearance measurements

CCX872 Significantly Decreased Glomerular Volume in Old BKS *db/db* Mice

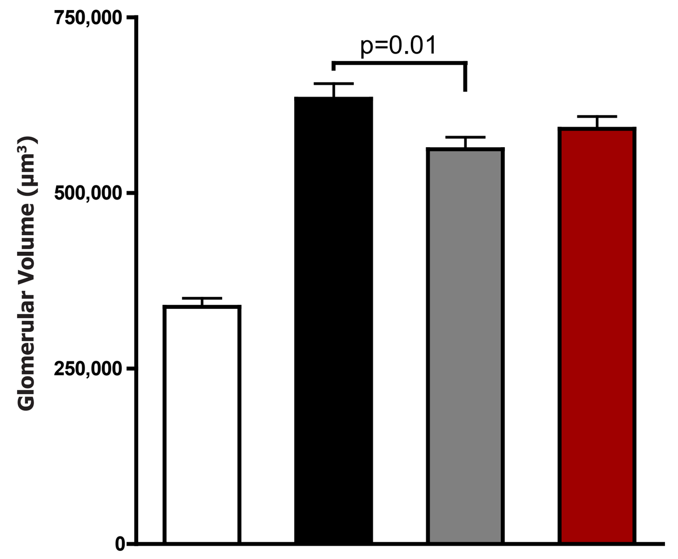

- 30 glomeruli analyzed from each kidney

CCX872 Significantly Increased Glomerular Podocyte Density in Old BKS *db/db* Mice

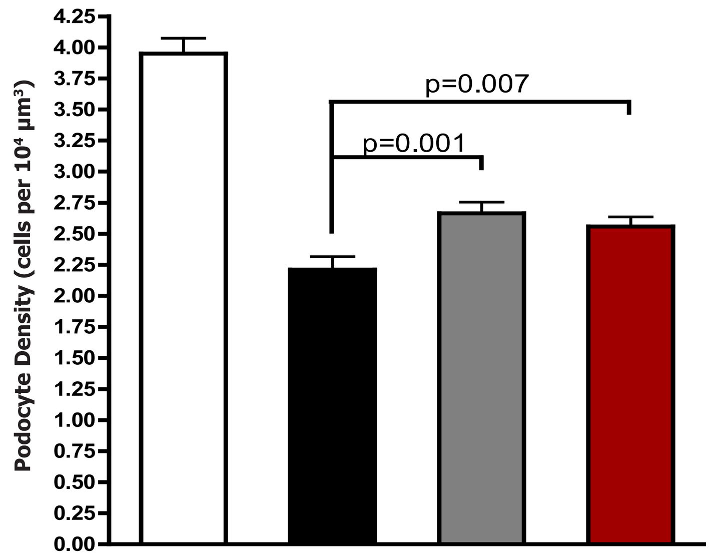

- 30 glomeruli analyzed from each kidney

WT normoglycemic | *db/db* Vehicle | *db/db* CCX872 (10 mg/kg) | *db/db* CCX872 (100 mg/kg)

Representative Images of Podocyte Nuclear WT-1 Immunohistochemistry

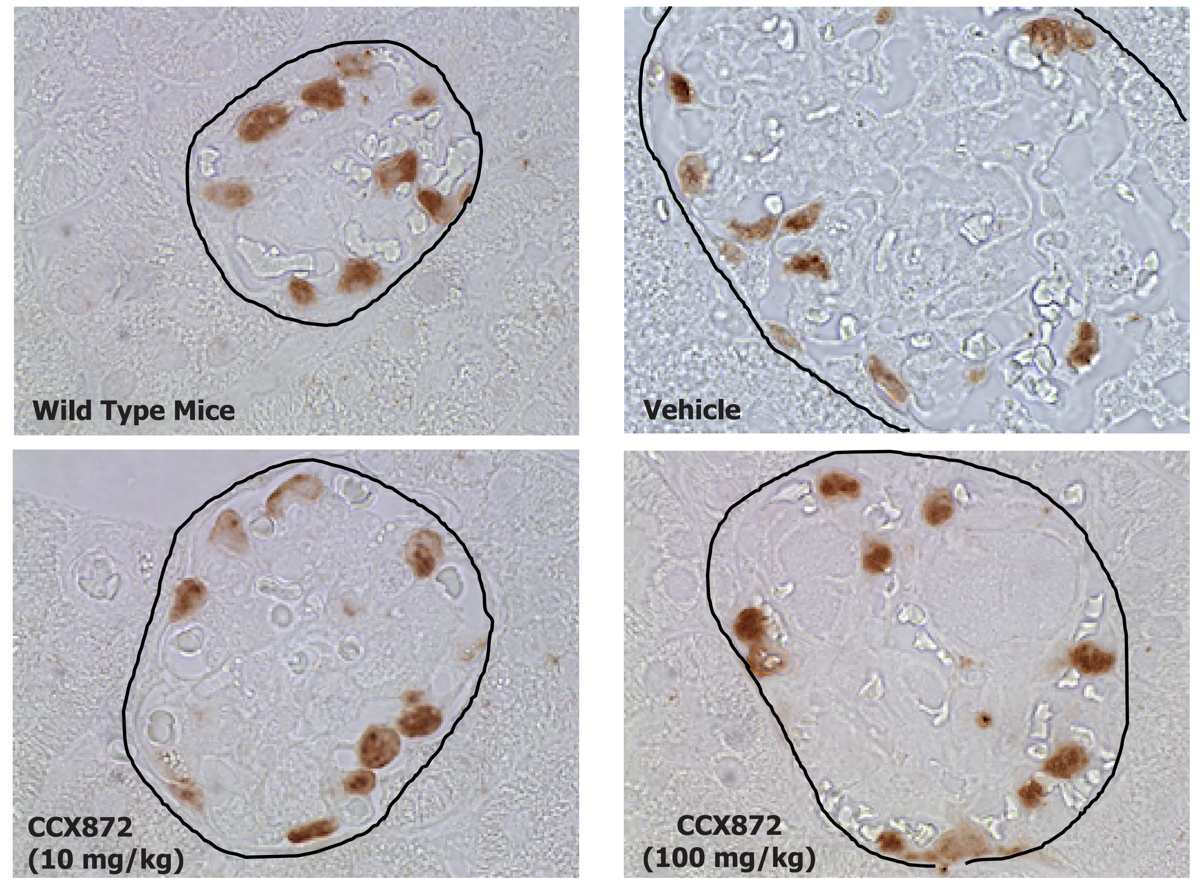

- Circles represent glomeruli; all images collected at the same magnification (400x).

CCX872 Significantly Reduced Interstitial F4/80+ Macrophage Content in Old BKS *db/db* Mice

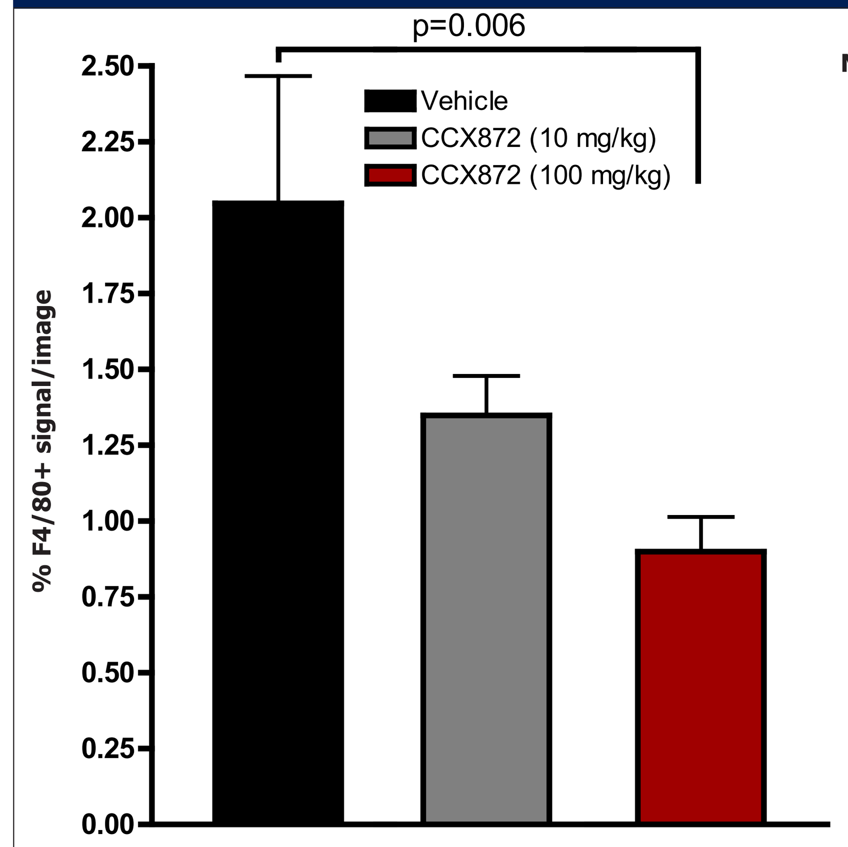

Representative Images of F4/80 Macrophage Immunohistochemistry

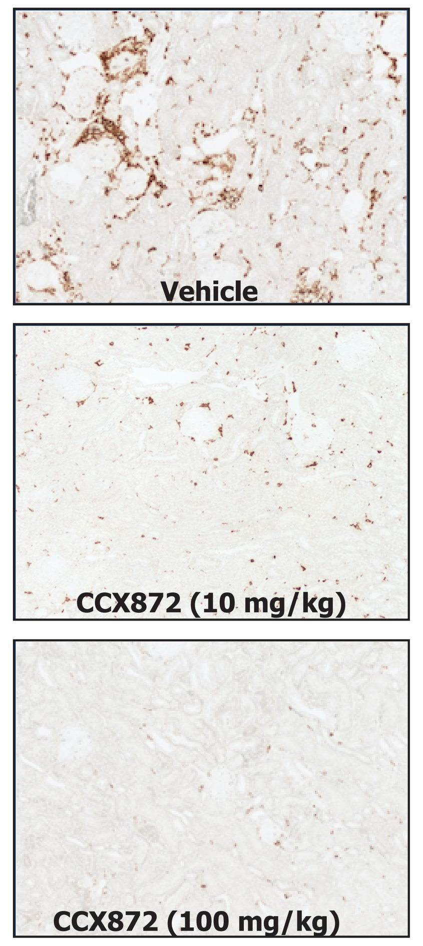

- 5-6 low power images from 2 depths analyzed per mouse. F4/80 signal was analyzed by software to give a % area that was positive for F4/80 signal.
- Very few F4/80 cells were detected in glomeruli.

CCX872 Decreases Glomerular 7/4+ Monocyte Numbers in Old BKS *db/db* Mice

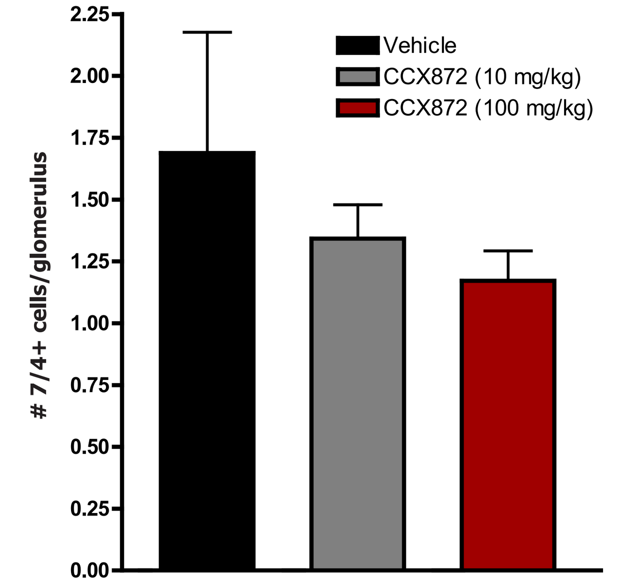

- 200-300 glomeruli analyzed from each kidney.

CCX872 Decreases Interstitial 7/4+ Monocyte Numbers in Old BKS *db/db* Mice

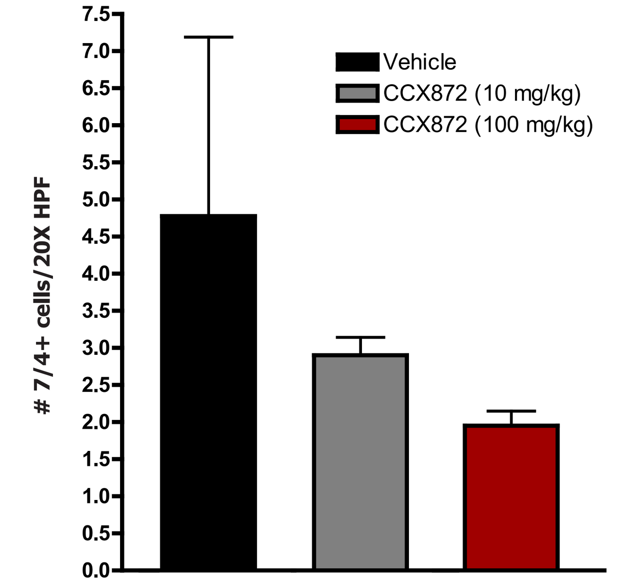

- Interstitial analysis based on 16-20 high power fields (20X) with blood vessels and glomeruli subtracted out.

CCX872 Decreases Glomerular CD3+ Lymphocyte Numbers in Old BKS *db/db* Mice

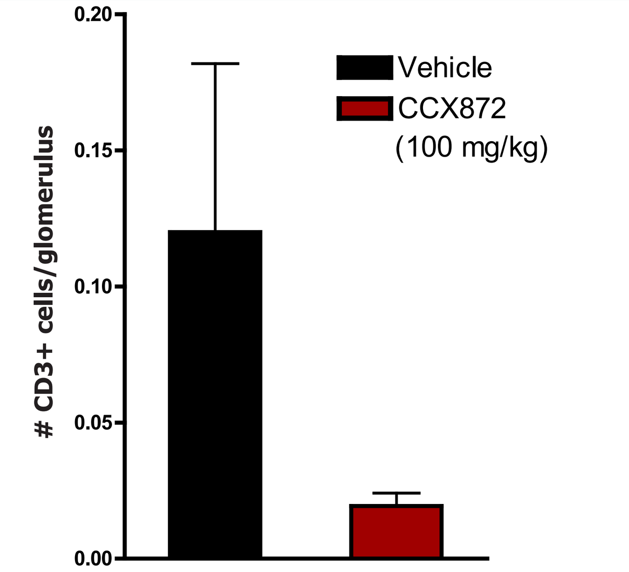

Representative Images of 7/4 Monocyte IHC

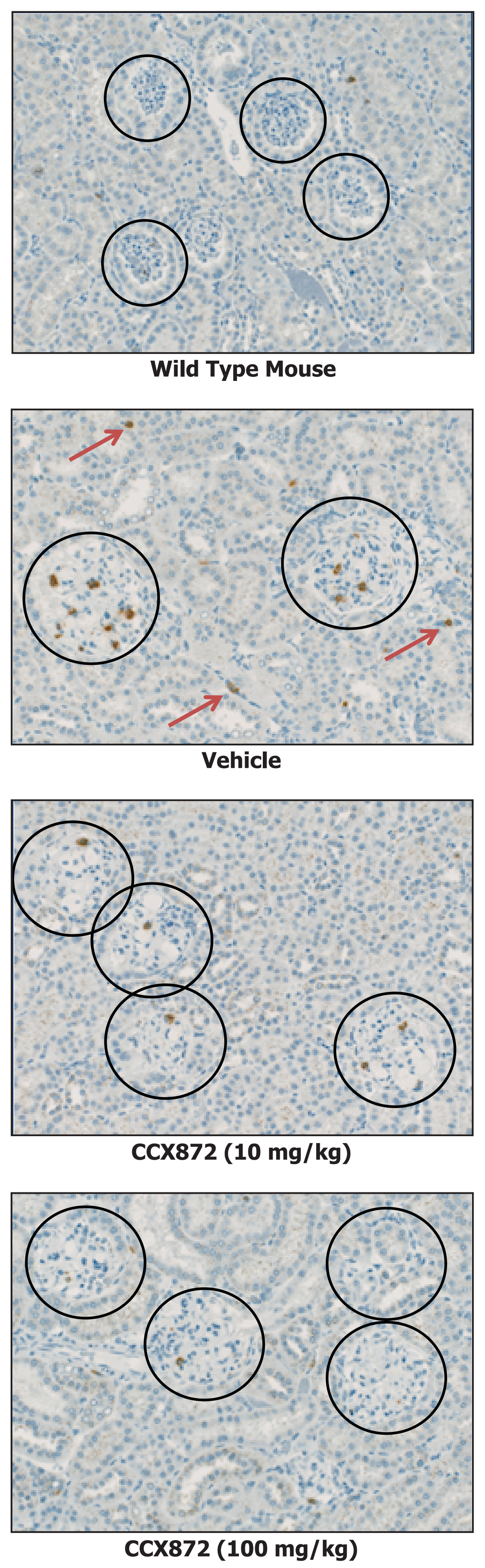

- Circles represent glomeruli. Red arrows point to the occasional interstitial 7/4+ cells found in *db/db* mice.

Summary and Conclusions

- The novel CCR2 antagonist CCX872, which is sufficiently potent on mouse CCR2 to serve as a useful pharmacological probe, was used in mouse models of diabetic nephropathy instead of the clinical candidate CCX140-B, which is selective for human CCR2 and is currently in two Phase 2 clinical trials in subjects with type 2 diabetic nephropathy.
- CCX872 doses and route of administration in mice were selected to produce systemic CCR2 coverage similar to that associated with clinical doses of CCX140-B.
- CCX872 significantly reduced total daily urinary albumin excretion (UAER) and urinary albumin:creatinine ratio (ACR) in 28-week old, diabetic BKS *db/db* mice with significant proteinuria at start of study.
- Effects were evident within 1 week of treatment and were maintained for the duration of the study (13 weeks).
- CCX872 treatment resulted in normalization of the glomerular hyperfiltration seen in these animals (using the inulin-FITC plasma clearance method).
- CCX872 significantly reduced glomerular hypertrophy and increased podocyte density.
- CCX872 reduced leukocyte infiltration into the kidney.
  - Significantly reduced interstitial F4/80 macrophage numbers.
  - Trend towards decreased glomerular 7/4+ monocytes as well as CD3+ lymphocytes in glomeruli and interstitium.

These data provide additional support for the ongoing clinical study of CCR2 antagonists such as CCX140-B in the treatment of diabetic nephropathy
